# E-Cadherin Immunohistochemical Expression in Gastrointestinal Adenocarcinomas and Its Association With Histological and Prognostic Parameters

**DOI:** 10.7759/cureus.101801

**Published:** 2026-01-18

**Authors:** Sarumathi Varadan, Uma Balasundararajan, Dhivya Manoharan, Balaji S Mahendran

**Affiliations:** 1 Department of Pathology, Indira Medical College and Hospital, Thiruvallur, IND; 2 Department of Pathology, K.A.P. Viswanatham Government Medical College, Trichy, IND; 3 Department of Community Medicine, Srinivasan Medical College and Hospital, Dhanalakshmi Srinivasan University, Trichy, IND

**Keywords:** adenocarcinoma, colorectal cancer, e-cadherin, gastric cancer, gastrointestinal

## Abstract

Background: E-cadherin (CDH1) is a critical cell adhesion molecule that maintains epithelial tissue integrity. Its loss or dysfunction is a hallmark of the epithelial-mesenchymal transition (EMT) and is mostly implicated in gastrointestinal adenocarcinomas, their invasion, and metastasis.

Objectives: This study aims to evaluate the patterns of E-cadherin immunohistochemical expression in gastrointestinal (GI) adenocarcinomas using a semi-quantitative scoring system and to correlate these patterns with the histological and prognostic factors of tumors.

Methods: This prospective and retrospective observational study included 50 patients diagnosed with GI adenocarcinoma (stomach, colon, and rectum) at a tertiary care center. Immunohistochemistry (IHC) for E-cadherin was performed on formalin-fixed paraffin-embedded tissue, and the expression of E-cadherin was evaluated using the Jawhari et al. scoring system. Statistical associations between expression patterns and clinicopathological variables were analyzed using the chi-square test, with a p-value < 0.05 considered significant.

Results: The study population (N=50) comprised 21 gastric (42%), 10 colonic (20%), and 19 rectal adenocarcinomas (38%). Reduced or absent staining of E-cadherin (score 0 - 1) was seen in 19 (38%) of GI adenocarcinomas, and 14 (28%) tumors showed completely preserved E-cadherin expression (score 3). Well-differentiated tumors showed preserved E-cadherin expression (scores 2-3 in all cases), whereas poorly differentiated tumors predominantly showed reduced or complete loss of expression (p=0.001). Adenocarcinoma NOS and intestinal-type tumors largely retained E-cadherin, while signet ring cell, mucinous, and infiltrating types frequently demonstrated markedly decreased expression (p=0.001). No statistically significant association was observed between tumor site and E-cadherin expression. Reduced or absent E-cadherin expression was significantly associated with advanced tumor stage (p=0.002), higher nodal stage (p=0.018), and perineural invasion (p=0.008).

Conclusion: E-cadherin dysregulation is strongly associated with histological tumor grade, adenocarcinoma type, tumor stage, nodal metastasis, and perineural invasion in GI adenocarcinomas. The significant loss of expression in poorly differentiated and infiltrating tumor types supports its role as an invasion suppressor. Loss of E-cadherin expression in advanced tumor stages, higher nodal metastasis, and perineural invasion elucidate the role of E-cadherin as a useful prognostic immunohistochemical marker in gastrointestinal adenocarcinomas.

## Introduction

Gastrointestinal (GI) cancers, encompassing malignancies of the stomach, colon, and rectum, represent a significant global health burden. Gastric cancers and colorectal cancers account for around 10% of all cancers in India and are the third most common type of cancer worldwide [1,2]. A pivotal mechanism in the progression and metastasis of these carcinomas is the disruption of cell-cell adhesion, a process often initiated by the dysfunction of the E-cadherin-catenin complex. E-cadherin (epithelial cadherin), encoded by the CDH1 gene on chromosome 16q22.1, is a calcium-dependent transmembrane glycoprotein that localizes to the adherens junctions of epithelial cells. It functions as a potent invasion suppressor; its extracellular domain mediates homotypic adhesion between adjacent cells, while its cytoplasmic tail interacts with beta-catenin and p120-catenin to anchor the complex to the actin cytoskeleton [3].

The loss of E-cadherin function is a hallmark of the Epithelial-Mesenchymal Transition (EMT), a biological process where polarized epithelial cells undergo multiple biochemical changes that enable them to assume a mesenchymal cell phenotype, which includes enhanced migratory capacity, invasiveness, and elevated resistance to apoptosis [4]. Mechanisms leading to E-cadherin inactivation include somatic mutations, promoter hypermethylation, transcriptional silencing by repressors such as Snail and Slug, and aberrant protein trafficking [5].

In histopathology, E-cadherin expression patterns serve as valuable biomarkers for tumor differentiation and aggressiveness. While normal epithelium shows strong membranous staining, carcinomas often exhibit heterogeneous patterns, cytoplasmic mislocalization, or complete loss. The semi-quantitative scoring system proposed by Jawhari et al. allows for a nuanced evaluation of these patterns, distinguishing between normal membranous staining, heterogeneous expression, cytoplasmic redistribution, and complete absence [6].

Previous literature has established a strong link between E-cadherin loss and diffuse-type gastric cancers [7] as well as poor prognosis in colorectal cancer [8]. However, comparative data analyzing the specific patterns of expression across different GI sites and their prognostic significance within the same cohort using the Jawhari method is less common. This study primarily aimed to evaluate the pattern of immunohistochemical expression of E-cadherin in GI adenocarcinomas. The secondary objective was to determine the association of E-cadherin expression with the histological and prognostic factors of GI adenocarcinomas.

## Materials and methods

Study design and setting

This ambispective observational single-center study was conducted at the Department of Pathology, Dhanalakshmi Srinivasan Medical College and Hospital, Perambalur. The study protocol was reviewed and approved by the Institutional Ethics Committee (Human Study) (IECHS) (Approval No: IECHS/IRCHS/DSMCH/Cert/012/2019, dated 17.10.2019).

Study population

The study population consisted of gastrointestinal tract lesion biopsies (endoscopic or surgical) received for histopathological examination in the Department of Pathology, DSMCH, from 2017 to 2022. All endoscopic and surgical biopsies diagnosed as adenocarcinoma of the gastrointestinal tract (stomach, colon, or rectum) on histopathological examination were included in the study. Cases were excluded if they were neoplastic lesions other than adenocarcinoma, had insufficient tissue in the blocks, or if the patients had received preoperative chemotherapy or radiotherapy.

Sample size

Based on the prevalence (p) of E-cadherin expression in gastrointestinal adenocarcinomas reported in Torabizadeh et al. [9] as 48.6%, with a precision (d) of 14% at a 95% confidence interval (Z₁₋ₐₗ₂ = 1.96), the sample size is calculated as N = Z²₁₋ₐₗ₂ p (1 - p) / d² = 48.9, which is rounded off as 50 cases.

Study procedure

Relevant clinical details of the patients whose GI adenocarcinoma tissue blocks were retrieved were collected prospectively as well as retrospectively, as per the availability of data. Formalin-fixed, paraffin-embedded tissue blocks were retrieved, and the diagnosis was confirmed based on morphological findings using Hematoxylin and Eosin (H&E) staining. E-cadherin immunohistochemistry was performed, and expression of e-cadherin on malignant cells in all study cases was evaluated using Jawhari et al. criteria.

Immunohistochemistry (IHC)

Immunohistochemistry was performed on formalin-fixed, paraffin-embedded (FFPE) tissue sections cut at 3 µm thickness and mounted on charged slides. The slides were incubated at 60-70°C for 1 hour to ensure optimal tissue adhesion. Sections were deparaffinized in two changes of xylene for 15 minutes each and rehydrated through descending grades of alcohol: absolute alcohol (two changes, 5 minutes each), 90% alcohol (5 minutes), and 70% alcohol (5 minutes), followed by two washes in distilled water (2 minutes each).

Antigen retrieval was performed using Tris‑EDTA buffer (Cat#PS009) at pH 9.0, using PathnSitu's MERS (Multi‑Epitope Retrieval System) under steam pressure for 15 minutes, followed by cooling to room temperature and two washes in distilled water and one wash in PBS/TBS. Endogenous peroxidase activity was blocked by applying hydrogen peroxide to the sections for 5 minutes, followed by two washes in wash buffer for 2 minutes each. E‑cadherin immunostaining was carried out using a rabbit monoclonal antibody, clone EP6 (PathnSitu Biotechnologies, Hyderabad, India), used as a ready‑to‑use reagent according to the manufacturer’s instructions. Sections were incubated with the primary antibody for 30 minutes in a moist chamber, then washed twice in wash buffer (2 minutes each).

Immunodetection was performed using the PathnSitu PolyExcel HRP polymer detection system. Polyexcel target binder reagent was added and incubated for 12 minutes, followed by two buffer washes (2 minutes each), after which Polyexcel HRP was applied and incubated for a further 12 minutes with two additional buffer washes (2 minutes each). Immunoreactivity was visualized using 3,3′-diaminobenzidine (DAB) chromogen, prepared by mixing 1 ml of DAB buffer with one drop of DAB chromogen, and incubated for 2-5 minutes. Sections were washed in distilled water and counterstained with hematoxylin for 30 seconds and then washed in water. Slides were then dehydrated through 70%, 90%, and absolute alcohol, cleared in xylene, and mounted in the usual manner.

Breast ductal carcinoma and normal colonic epithelium served as positive controls for E-cadherin, while negative controls were prepared by omission of the primary antibody. E-cadherin expression was assessed as membranous staining in tumor cells and interpreted in conjunction with appropriate controls.

IHC E-cadherin expression scoring system

E-cadherin expression was evaluated using the semi-quantitative scoring system described by Jawhari et al. [6]. Staining was classified into four scores: Score 0: Absent staining; Score 1: Cytoplasmic distribution (aberrant); Score 2: Heterogeneous staining (mixed cytoplasmic and membranous); Score 3: Normal membranous pattern of staining.

All immunohistochemically stained slides were independently evaluated by two experienced pathologists who were blinded to the clinical and histopathological details. In cases of discrepancy, a consensus score was arrived at after joint review.

Statistical analysis

Data was entered into Microsoft Excel and analyzed using IBM Corp. Released 2015. IBM SPSS Statistics for Windows, Version 21. Armonk, NY: IBM Corp. Descriptive statistics of demographic data and tumor characteristics were represented in frequencies and percentages. The chi-square test was employed to determine the association between E-cadherin expression and categorical variables (tumor site, histological grade, adenocarcinoma type, and prognostic factors), but Fisher's exact test was used when the expected values of the cells were less than 5. A p-value of < 0.05 was considered statistically significant.

## Results

Demographic and tumor characteristics

The study included 50 patients ranging in age from 31 to over 70 years. The majority of patients were in the 41-50 years (30%, n = 15) and 51-60 years (28%, n = 14) age groups. Males constituted 72% (n=36) of the study population, while females constituted 28% (n=14). The anatomical distribution of the tumors was stomach: 21 cases (42%), colon: 10 cases (20%), and rectum: 19 cases (38%). Histologically, the most common subtype was adenocarcinoma not otherwise specified (NOS) (30%, n = 15), followed by infiltrating adenocarcinoma (20%, n = 10). In terms of differentiation, 38% (n = 19) of tumors were well-differentiated, 30% (n = 15) moderately differentiated, and 32% (n = 16) poorly differentiated. The demographic characteristics of the patients and their tumor characteristics are elucidated in Table 1.

**Table 1:** Demographic and tumor characteristics

Demographic & tumor characteristics		Frequency	Percent
Age group	31 - 40 years	6	12%
	41 - 50 years	15	30%
	51 - 60 years	14	28%
	61 - 70 years	12	24%
	> 70 years	3	6%
Gender	Males	36	72%
	Females	14	28%
Procedure	Surgical biopsy	24	48%
	Endoscopic biopsy	26	52%
Tumor site	Stomach	21	42%
	Colon	10	20%
	Rectum	19	38%
Adenocarcinoma type	Adenocarcinoma NOS	15	30%
	Diffuse type	4	8%
	Intestinal type	5	10%
	Mixed type	7	14%
	Mucinous type	2	4%
	Signet Ring type	7	14%
	Infiltrating	10	20%
Histological Grade	Well differentiated	19	38%
	Moderately differentiated	15	30%
	Poorly differentiated	16	32%

E-cadherin expression in gastrointestinal adenocarcinomas

**Figure 1:**
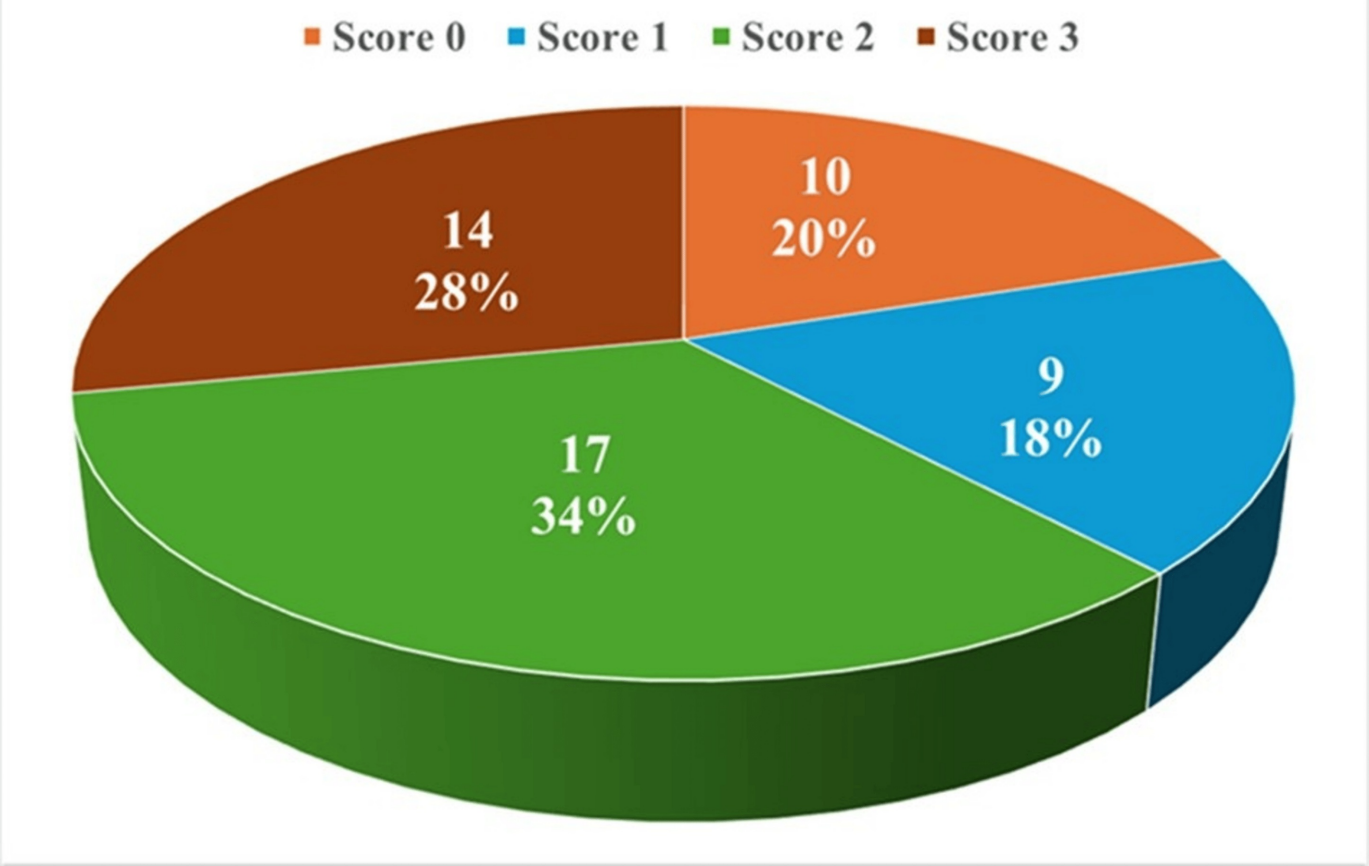
E-cadherin expression of tumors (Jawhari scoring) Score 0: Absent staining; Score 1: Cytoplasmic distribution (aberrant); Score 2: Heterogeneous staining (mixed cytoplasmic and membranous); Score 3: Normal membranous pattern of staining.

The distribution of E-cadherin expression in tumors, as assessed by Jawhari scoring, is visualized in Figure 1. A score of 2 was observed most frequently, comprising 17 cases (34%), while score 3 was present in 14 cases (28%), indicating relatively preserved E-cadherin expression in these tumors. Reduced expression was seen in 10 cases (20%) with a score of 0 and in 9 cases (18%) with a score of 1, representing a substantial subset of tumors with diminished or lost E-cadherin staining.

**Figure 2:**
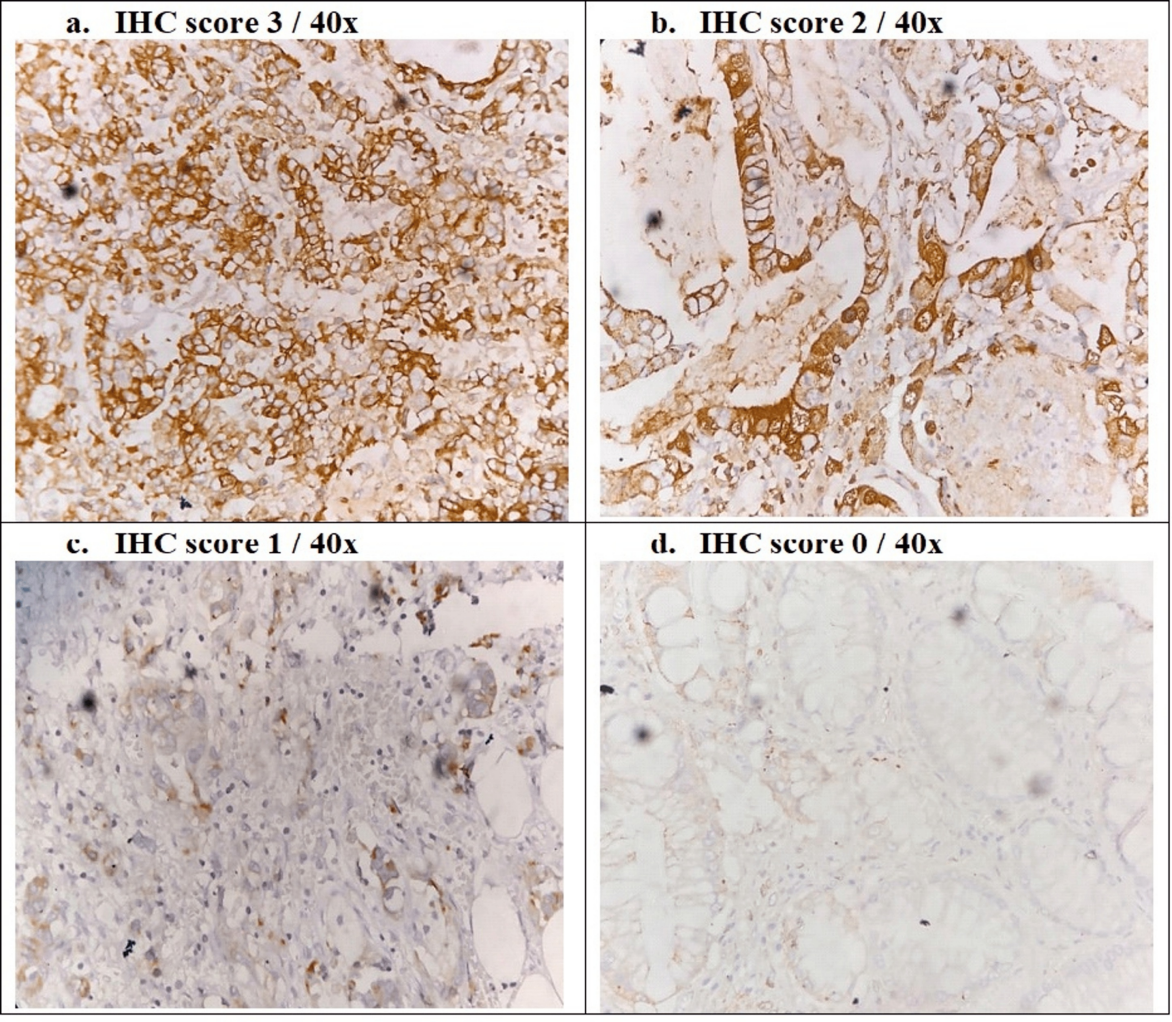
E-cadherin IHC staining a: strong membranous staining - score 3 b: cytoplasmic and membranous staining - score 2 c: reduced cytoplasmic staining - score 1 d: faint or absent staining - score 0

The IHC images of the E-cadherin staining of different scores are visualized in Figure 2.

E-cadherin expression by tumor site

The patterns of E-cadherin expression varied across the three anatomical sites (Table 2). In gastric adenocarcinomas, 38% (n = 8/21) of cases retained normal membranous staining (score 3). However, a significant portion showed severe dysregulation, with 38% (n = 8/21) showing complete absence (score 0) or cytoplasmic staining (score 1). Colon tumors showed a distinct profile characterized by high heterogeneity, with 70% (n = 7/10) of cases with score 2, and notably, no cases showed complete loss (score 0). Rectal tumors exhibited the most aggressive loss of adhesion protein expression among the three sites, with the highest proportion of 52.6% (n = 10/19) with scores of 0 - 1. Only 21.1% (n=4/10) of rectal tumors retained normal membranous staining. However, the E-cadherin expression across different tumor sites was not statistically significant (p = 0.177).

**Table 2:** E-cadherin expression with tumor sites p-value by Fisher's Exact test (χ^2 ^= 9.85, df = 6)

Tumor site	E-cadherin expression								p-value
	0		1		2		3		
	n	%	n	%	n	%	n	%	
Stomach	4	19%	4	19%	5	23.8%	8	38.1%	0.177
Colon	0	0%	1	10%	7	70%	2	20%	
Rectum	6	31.6%	4	21.1%	5	26.3%	4	21.1%	

E-cadherin expression by histological grade

A highly significant association was observed between E-cadherin expression and the histological grade of differentiation (p = 0.001) (Table 3). E-cadherin expression was largely preserved in well-differentiated tumors, with no cases showing absent or purely cytoplasmic staining. All cases were either Score 2 (52.6%, n = 10/19) or Score 3 (47.4%, n = 9/19). Poorly differentiated tumors showed profound E-cadherin dysregulation. 50% (n = 8/16) of cases had complete loss of expression (score 0), and 37.5% (n = 6/16) showed aberrant cytoplasmic staining (score 1). Only 6.3% (n = 1/16) retained normal membranous staining (score 3). Moderately differentiated tumors had a varied distribution of E-cadherin expression from normal to completely aberrant.

**Table 3:** E-cadherin expression with histological grade p-value by Fisher's Exact test (χ2 = 29.15, df = 6)

Histological grade	E-cadherin expression								p-value
	0		1		2		3		
	n	%	n	%	n	%	n	%	
Well differentiated	0	0%	0	0%	10	52.6%	9	47.4%	0.001
Moderately differentiated	2	13.3%	3	20%	6	40%	4	26.7%	
Poorly differentiated	8	50%	6	37.5%	1	6.3%	1	6.3%	

E-cadherin expression by adenocarcinoma type

The specific histological subtypes of adenocarcinomas also showed distinct expression patterns (p = 0.001) (Table 4). Among the intestinal type of tumors, 80% (n = 4/5) retained a score of 3 (normal), consistent with their cohesive morphology. Adenocarcinomas of the NOS type also showed similarity to the intestinal type, with 47% (n = 7/15) of score 3 and 53% (n = 8/15) of score 2. The diffuse-type tumors showed significant loss of E-cadherin in 50% (n = 2/4) with scores of 0-1. Similar to the diffuse type, Signet Ring Cell type tumors also showed high dysregulation in 57.1% (n = 4/7) with scores of 0 - 1. Infiltrating adenocarcinomas showed the most severe profile, with 50% (n = 5/10) scoring 0 and 40% (n = 4/10) scoring 1, indicating near-total dysfunction of the adhesion complex. Mixed and mucinous types had a varied distribution of E-cadherin expression.

**Table 4:** E-cadherin expression with adenocarcinoma type p-value by Fisher's Exact test (χ2 = 36.18, df = 18)

Adenocarcinoma type	E-cadherin expression								p-value
	0		1		2		3		
	n	%	n	%	n	%	n	%	
NOS	0	0%	0	0%	8	53.3%	7	46.7%	0.001
Diffuse type	1	25%	1	25%	2	50%	0	0 %	
Intestinal type	0	0%	0	0%	1	20%	4	80%	
Mixed type	1	14.3%	2	28.6%	1	14.3%	3	42.9%	
Mucinous type	1	50%	0	0%	1	50%	0	0%	
Signet Ring type	2	28.6%	2	28.6%	3	42.9%	0	0%	
Infiltrating	5	50%	4	40%	1	10%	0	0%	

E-cadherin expression with prognostic factors

Among the prognostic factors analyzed only with surgical biopsies (n = 24), E‑cadherin expression showed a significant inverse association with advancing tumor stage and adverse pathological features (Table 5). E-cadherin expression showed a significant inverse association with advancing tumor stage, with reduced or absent expression predominantly observed in advanced tumors (pT3 and above), while preserved expression was confined to early-stage tumors (pT2 and below) (p = 0.002). A similar significant association was noted with nodal metastasis, wherein higher nodal stage (pN2 and above) correlated with lower E-cadherin expression (p = 0.018). Tumors exhibiting perineural invasion demonstrated significantly reduced E-cadherin expression compared to those without perineural invasion (p = 0.008). Although reduced E-cadherin expression was more frequent in cases with lymphovascular invasion, this association did not reach statistical significance (p = 0.216). These findings indicate that loss of E‑cadherin expression parallels increasing tumor aggressiveness and poorer prognostic parameters in gastrointestinal adenocarcinomas.

**Table 5:** E-cadherin expression with prognostic factors p-values by Fisher's Exact test: tumor stage (χ2 = 12.44, df = 3); Nodal metastasis (χ2 = 9.54, df = 3); lymphovascular invasion (χ2 = 4.73, df = 3); perineural invasion (χ2 = 11.44, df = 3)

Prognostic factors		E-cadherin expression								p-value
		0		1		2		3		
		n	%	n	%	n	%	n	%	
Tumor stage	pT2 & below	0	0.0%	1	33.3%	4	44.4%	7	100.0%	0.002
	pT3 & above	5	100.0%	2	66.7%	5	55.6%	0	0.0%	
Nodal Metastasis	pN1 & below	1	20.0%	1	33.3%	8	88.9%	6	85.7%	0.018
	pN2 & above	4	80.0%	2	66.7%	1	11.1%	1	14.3%	
Lympho vascular Invasion	Present	3	60.0%	1	33.3%	1	11.1%	1	14.3%	0.216
	Absent	2	40.0%	2	66.7%	8	88.9%	6	85.7%	
Perineural Invasion	Present	4	80.0%	1	33.3%	1	11.1%	0	0.0%	0.008
	Absent	1	20.0%	2	66.7%	8	88.9%	7	100.0%	

## Discussion

E‑cadherin, a calcium‑dependent adhesion molecule encoded by the CDH1 gene, is pivotal for the maintenance of epithelial polarity, glandular architecture, and suppression of epithelial-mesenchymal transition in gastrointestinal tissues. In the present study, gastrointestinal adenocarcinomas showed a heterogeneous pattern of E‑cadherin expression, with 62% of the tumors retaining preserved membranous staining (Jawhari scores 2-3) and the remainder, 38%, exhibiting reduced or complete loss of expression. This overall proportion of abnormal expression is comparable to several gastric and colorectal series, in which aberrant or decreased E‑cadherin immunostaining has been reported in 40-60% of cases, highlighting that disruption of this adhesion system is a frequent event in gastrointestinal carcinogenesis [10-12].

The results of this study reaffirm the critical role of E-cadherin in maintaining the epithelial phenotype and demonstrate that its loss is a key event in the de-differentiation of gastrointestinal adenocarcinomas. The most striking finding in our study is the statistically significant inverse association between tumor grade and E-cadherin scores. In well-differentiated tumors, the adhesion complex was preserved (scores 2 and 3), maintaining glandular architecture. Conversely, 87.5% of poorly differentiated tumors exhibited severe dysregulation (scores 0 and 1). This aligns with the findings of Sadanandan et al. [13], a study that specifically found that 100% of poorly differentiated tumors had aberrant E-cadherin staining, compared to only 12.5% of well-differentiated tumors in gastric cancers. Lazar et al. [14] and Kanazawa et al. [5] also reported about 61.5% and 80% of poorly differentiated tumors showing aberrant E-cadherin staining in gastric and colorectal cancers, respectively. Mayer et al. [15] and others also have documented that downregulation of E-cadherin correlates with cellular de-differentiation. The shift from membranous (score 3) to cytoplasmic (score 1) or absent (score 0) staining represents the molecular basis for the loss of tissue architecture seen in high-grade tumors.

In the present cohort, no statistically significant association was observed between anatomical site (stomach, colon, or rectum) and E‑cadherin expression, with all three locations showing a mixture of preserved and reduced staining across Jawhari categories. This suggests that alterations in E‑cadherin are driven more by tumor‑intrinsic molecular events than by regional differences in the gastrointestinal tract, a view consistent with molecular data indicating that CDH1 disruption and related signalling abnormalities can arise at multiple levels of the digestive epithelium [16]. Previous site‑specific studies have variably reported higher frequencies of loss in diffuse gastric cancers or right‑sided colorectal cancers, but meta‑analyses emphasize that within each organ, expression correlates far more strongly with histological grade, invasion depth, and nodal status than with topography [10,11]. The absence of a site‑wise difference in this study, therefore, agrees with the broader notion that E‑cadherin status reflects aggressive biological behavior rather than simple anatomical location.

When adenocarcinomas were subclassified by histological type, adenocarcinoma not otherwise specified (NOS) and intestinal‑type tumors predominantly retained strong E‑cadherin expression, whereas signet‑ring, mucinous, diffuse, mixed, and infiltrating types demonstrated much higher proportions of reduced or absent staining. In particular, infiltrating and signet‑ring carcinomas showed frequent scores of 0-1, indicating near‑complete loss of membranous E‑cadherin in a large subset of cases. This pattern closely parallels the classical observations of Jawhari and colleagues, who described abnormal E‑cadherin-catenin complex immunoreactivity in diffuse and poorly cohesive gastric carcinomas and related this to discohesive growth and stromal infiltration [6]. The study reflected that among the gastric cancers, intestinal-type tumors retained E-cadherin expression, while diffuse and signet-ring types showed reduced expression of E-cadherin. This supports the established "two-hit" theory in diffuse gastric cancer, involving CDH1 mutation and promoter methylation, leading to aberrant protein localization, changes that collectively drive loss of cohesive behavior and early serosal or peritoneal spread [17]. Multiple studies provide robust evidence for this finding. Divyagna et al. [18] found membranous E-cadherin staining in 85% of intestinal-type cancers, compared to only 30% in diffuse-type cancers. Karayiannakis et al. [19] similarly reported abnormal E-cadherin expression in 96% of diffuse tumors versus 47% of intestinal-type tumors. Similar trends have been reported in signet‑ring cell and mucinous colorectal carcinomas, where markedly reduced E‑cadherin expression correlates with aggressive clinical behavior and inferior survival compared with conventional gland‑forming adenocarcinomas [20]. The infiltrating adenocarcinoma subtype in our study showed the most severe E-cadherin loss (90% score 0 or 1). This morphology corresponds to the "tumor budding" phenomenon often described in colorectal cancer, where cells at the invasive front undergo EMT, lose E-cadherin, and migrate singly or in small clusters [21]. The strong association between histological subtype and expression profile in the present study is therefore in keeping with the well‑established link between E‑cadherin loss and discohesive variants across the gastrointestinal tract.

In the present study, reduced or absent E-cadherin expression was significantly associated with advanced tumor stage (pT3 & above) and higher nodal metastasis (pN2 & above), which is consistent with the recognized role of E-cadherin loss in promoting tumor invasion and metastatic dissemination in gastrointestinal malignancies. These findings align with observations in colorectal adenocarcinoma, where low E-cadherin expression correlated with higher T stage and lymph node metastasis, reflecting epithelial-mesenchymal transition and aggressive behavior as reported in previous IHC studies of colorectal cancer patients [22]. Similarly, meta-analyses in gastric cancer have demonstrated that reduced E-cadherin expression is associated with advanced TNM stage, deeper invasion, lymph node metastasis, and poor overall survival, reinforcing its clinicopathological significance in GI adenocarcinomas [23]. The significant association between E-cadherin loss and perineural invasion in our cohort further corroborates data from large series where negative E-cadherin status was an independent adverse prognostic indicator, including associations with perineural and vascular invasion. Although lymphovascular invasion did not reach statistical significance in our study, this trend of reduced E-cadherin expression in more invasive phenotypes mirrors the broader literature suggesting its role in tumor progression and poorer outcomes, as highlighted in meta-analytic data [8].

From a mechanistic perspective, the patterns observed in this study are compatible with the multifactorial regulation of E‑cadherin in gastrointestinal malignancies. CDH1 can be inactivated through germline or somatic mutations, promoter hypermethylation, transcriptional repression by EMT‑inducing transcription factors, and post‑translational changes affecting protein trafficking or stability [24]. Diffuse gastric cancer and some signet‑ring carcinomas of the stomach and colon are paradigmatic examples in which CDH1 mutations and epigenetic silencing drive near‑complete loss of membranous E‑cadherin, leading to single‑cell infiltration and linitis plastica‑like morphology [25,26]. Conversely, intestinal‑type and conventional colorectal adenocarcinomas more often display preserved expression, with focal reduction emerging late in tumor progression and correlating with budding, invasion front changes, and nodal metastasis.

The differential expression patterns across histological subtypes in this series fit well with these mechanistic models, suggesting that the immunohistochemical profile captured by Jawhari scoring reflects underlying genetic and epigenetic events. The consistent association in this study between low E‑cadherin expression and both poor differentiation and aggressive histological subtypes supports its potential use as part of a multiparametric panel for risk stratification. In practice, identifying tumors with scores 0-1 could help flag cases deserving closer surveillance, consideration of more intensive adjuvant therapy, or inclusion in trials evaluating EMT‑targeted interventions.

Finally, the strengths and limitations of the present work should be acknowledged. The use of a standardized and widely validated immunohistochemical scoring system (Jawhari scoring) enhances the reproducibility and comparability of findings with previously published studies. Inclusion of multiple gastrointestinal sites and diverse histological subtypes allowed meaningful comparison across tumor phenotypes and reinforced the concept that E-cadherin loss reflects tumor aggressiveness rather than anatomical location alone. Furthermore, the statistically significant associations observed with key adverse prognostic factors strengthen the biological plausibility of E-cadherin as a marker of tumor progression and epithelial-mesenchymal transition. The sample size, although adequate for detecting significant associations, may limit the power of subgroup analyses, particularly for less common histological variants such as infiltrating or signet-ring carcinomas. As a cross-sectional, histopathology-based study, long-term clinical outcomes such as disease-free or overall survival could not be assessed, precluding direct prognostic validation. Additionally, while inter-observer consistency was addressed through blinded evaluation and consensus scoring, some degree of subjectivity inherent to semi-quantitative IHC assessment cannot be completely eliminated. Future studies integrating E‑cadherin expression with detailed molecular profiling, tumor budding assessment, and clinical outcomes could further clarify its role in prognostic algorithms and in guiding targeted or personalized therapeutic strategies in gastrointestinal adenocarcinomas.

## Conclusions

The present study demonstrates that E-cadherin expression in gastrointestinal adenocarcinomas is heterogeneous and frequently altered, reflecting its pivotal role in maintaining epithelial integrity and suppressing tumor progression. Reduced or complete loss of membranous E-cadherin expression was significantly associated with adverse histopathological and prognostic parameters, including poor tumor differentiation, advanced tumor stage, higher nodal metastasis, and the presence of perineural invasion. Aggressive histological subtypes such as diffuse, signet-ring, mucinous, and infiltrating adenocarcinomas showed a marked tendency toward aberrant E-cadherin expression, whereas well-differentiated and intestinal-type tumors largely retained preserved membranous staining. These findings support the concept that E-cadherin loss is a key event in epithelial-mesenchymal transition and tumor de-differentiation, contributing to increased invasiveness and metastatic potential in gastrointestinal malignancies. Overall, this study highlights the potential utility of E-cadherin as a prognostic immunohistochemical biomarker, which, when interpreted alongside conventional histopathological parameters, may aid in risk stratification and identification of biologically aggressive gastrointestinal adenocarcinomas. Future studies incorporating molecular analyses and long-term clinical outcomes are warranted to further validate its prognostic and therapeutic implications.
